# Baseline Mapping of Neglected Tropical Diseases in Africa: The Accelerated WHO/AFRO Mapping Project

**DOI:** 10.4269/ajtmh.20-1538

**Published:** 2021-04-26

**Authors:** Maria P. Rebollo, Adiele Nkasiobi Onyeze, Alexandre Tiendrebeogo, Mutale Nsakashalo Senkwe, Benido Impouma, Kisito Ogoussan, Honorat G. M. Zouré, Kebede Deribe, Jorge Cano, Ekoue Boniface Kinvi, Andrew Majewski, Eric A. Ottesen, Patrick Lammie

**Affiliations:** 1 Expanded Special Project for Elimination of NTDs, World Health Organization Regional Office for Africa, Brazzaville, Republic of Congo;; 2 IST for Eastern and Southern Africa, World Health Organization, Harare, Zimbabwe;; 3 World Health Organization Regional Office for Africa, Brazzaville, Republic of Congo;; 4 World Health Organization, Country Office, Juba, South Sudan;; 5 FHI 360, Washington, District of Columbia;; 6 Brighton and Sussex Medical School, Brighton, United Kingdom;; 7 The Taskforce for Global Health, Decatur, Georgia

## Abstract

Mapping is a prerequisite for effective implementation of interventions against neglected tropical diseases (NTDs). Before the accelerated World Health Organization (WHO)/Regional Office for Africa (AFRO) NTD Mapping Project was initiated in 2014, mapping efforts in many countries were frequently carried out in an ad hoc and nonstandardized fashion. In 2013, there were at least 2,200 different districts (of the 4,851 districts in the WHO African region) that still required mapping, and in many of these districts, more than one disease needed to be mapped. During its 3-year duration from January 2014 through the end of 2016, the project carried out mapping surveys for one or more NTDs in at least 2,500 districts in 37 African countries. At the end of 2016, most (90%) of the 4,851 districts had completed the WHO-required mapping surveys for the five targeted Preventive Chemotherapy (PC)-NTDs, and the impact of this accelerated WHO/AFRO NTD Mapping Project proved to be much greater than just the detailed mapping results themselves. Indeed, the AFRO Mapping Project dramatically energized and empowered national NTD programs, attracted donor support for expanding these programs, and developed both a robust NTD mapping database and data portal. By clarifying the prevalence and burden of NTDs, the project provided not only the metrics and technical framework for guiding and tracking program implementation and success but also the research opportunities for developing improved diagnostic and epidemiologic sampling tools for all 5 PC-NTDs—lymphatic filariasis, onchocerciasis, schistosomiasis, soil-transmitted helminthiasis, and trachoma.

## INTRODUCTION

Mapping is an essential element for each of the neglected tropical disease (NTD) programs advocated by the WHO. It consists of the epidemiological surveys that provide baseline information on a disease’s geographical distribution, the level of disease endemicity, and the populations or age-groups in need of preventive chemotherapy (PC)—all of which information is critical for planning, implementing, monitoring, and evaluating NTD programs.^1,2^ Indeed, the appropriate targeting of mass drug distribution (MDA) requires an understanding of the geographical distribution of NTDs sufficient to identify those areas that need the MDA interventions and those that do not, based on WHO guidelines. Without such data, prioritization of treatment initiatives must default to expert opinion or outdated observations collected historically—potentially leading not only to under-treatment of needy populations but also to treatment in areas where treatment is not required and, thus, to wastage of donated medicines, time, and other precious resources.

As early as 2005, the WHO began to categorize a group of diseases as “NTDs”^3^ and to reorganize its offices in Geneva at that time to create a department targeting the NTDs. For approaching these NTDs, the concept of “geographic mapping for prevalence” was first expanded to “geographic mapping for action”; that is, identifying not just *where* the diseases are found but also the specific programmatic treatment or implementation actions that were required to target each area where one or more NTDs are present.^4,5^ In the WHO African region, the challenge of “action mapping” for the NTDs was especially formidable. First, the region comprised a population of nearly one billion people in 47 countries spread over a vast geographical area.^6^ Second, “action mapping” required that for each of the NTDs that needed to be action-mapped, defined program targets or strategies must exist to which specific actions could be linked—actions determined partly by the disease’s pre-intervention, baseline prevalence, and partly by the prior activities undertaken by the programs themselves.^7^

Between 2005 and 2012, progress of these mapping efforts was steady but slow. Then came the London Declaration on NTDs^8^ in 2012. At that event, the WHO, other international agencies, donor pharmaceutical companies, the Bill & Melinda Gates Foundation (BMGF), international and national non-governmental organizations (NGOs), and key bilateral development agencies all pledged to accelerate efforts targeting NTD elimination or high-level control. For each of these concerned partners, “action mapping” became an urgent priority. It was this opportune moment of enhanced public- and private-sector attention that enabled an accelerated, expanded “WHO/AFRO NTD Mapping Project” to be created: a 3-year project between late 2013 and 2016 that dramatically transformed the national NTD programs of the African region.

## MATERIALS AND METHODS

### Mapping standards and tools.

Before the WHO/AFRO Accelerated NTD Mapping Project was initiated in late 2013, mapping efforts had been carried out in many countries, but often in an *ad hoc*, non-standardized manner with data residing in a variety of locations. The accelerated mapping project, therefore, established standards and guidelines required for these NTD mapping surveys; specifically, agreement that 1) the targeted focus for this mapping would be the district or “admin 3” (which also usually serves as an “implementation unit” [IU] for NTD programs, defining those areas where decisions to determine programmatic treatment [or not] would be made), 2) use would be made of the WHO-agreed, standardized mapping methods to identify the prevalence of each of the five PC-NTDs in every district of every country, and 3) data would be collected and accessible centrally. Although additional, more refined assessments might later be required for specific programmatic needs^9^ or to address the known limitations of existing diagnostic tools in low-prevalence settings, and the principal goal of the accelerated mapping project was to ensure that PC-NTD endemicity status would be defined broadly for every district in AFRO’s 47 countries.

For lymphatic filariasis (LF), schistosomiasis (SCH), and soil-transmitted helminthiasis (STH), the mapping implementation followed published WHO guidelines and objectives^7,10,11^ and was carried out by technical teams of national program workers guided by regional and international consultants, including members of the WHO/AFRO’s NTD Expert Committee (the Regional Programme Review Group). Funding support came principally from the BMGF, national ministries of health, the international development agencies (the U.S. Agency for International Development [USAID] and the UK Department for International Development [DfID]), and numerous NGOs. The mapping teams were trained by expert consultants appointed by both WHO–AFRO and the NTD-Support Center (NTD-SC) at the Task Force for Global Health.

For onchocerciasis, because mapping had traditionally been the responsibility of the Onchocerciasis Control Programme (1974–2002) and African Programme for Onchocerciasis Control (APOC, 1996–2015), WHO/AFRO allocated a specific portion of the mapping project funds to APOC to complete “standard” onchocerciasis mapping^9^ where still necessary.

For trachoma, separate funds, principally from the DfID and USAID, were used to create the Global Trachoma Mapping Project^10^ that took responsibility for trachoma mapping in Africa during the same period when the accelerated WHO/AFRO NTD Mapping Project was active.

### Implementation strategy.

The overall strategy of the accelerated mapping project was, first, to determine the mapping gaps by collecting all historical information available on PC-NTD distribution and prevalence in the African Region and then to support large-scale programmatic implementation of needed mapping surveys using the most effective tools and approaches available to capture and record the data. The director of the mapping project (in Brazzaville) was supported by a regional NTD mapping coordinator and subregional NTD program managers in each of the West, Central, and East/South Africa subregions, along with a data manager and two administrative personnel. This team, with input from regional and global experts, designed and oversaw all planning for the implementation of the mapping activities.

Given the complexities of this challenge—first, the different numbers of different diseases needing to be mapped in different districts in different countries; second, the fact that unmapped districts had remained unmapped largely because of their remoteness, minimal infrastructure, conflict, and insecurity; third, the requirement for specific epidemiologic tools, diagnostics, and expertise to be available at each mapping site for the prevalence of the PC-NTDs to be determined accurately—this whole exercise presented a truly enormous organizational and logistical challenge. In total, almost 200 individuals were trained in the mapping itself, with another 50 individuals acting in various supervisory roles and literally hundreds of supporting field-workers. A further collaboration between WHO/AFRO and the NTD-SC at the Task Force for Global Health assisted with both technical management and provision of diagnostic tests, ancillary supplies, data management, and logistics efforts.

### Data capture and management.

The first activity of the accelerated NTD mapping project was to take stock of the available PC-NTD prevalence data still scattered in databases kept by WHO/AFRO, WHO/Geneva, APOC, national programs, and NGOs. The available data were categorized as being sourced from: 1) records of reported endemicity—that is, areas where the specific NTDs had been known or thought to be prevalent and where, on that basis, MDA treatment had *already* been initiated; 2) available survey data from earlier, actual mapping surveys—recorded in a variety of ways that still needed to be harmonized and collated; and 3) new surveys conducted during the life of the mapping project itself.

These three different kinds of data varied not only as to their *source* but also in the level and granularity at which they were collected, including district, subdistrict, village, site, specific population, and diagnostic tool. Decision rules were established on how to weight or prioritize related data to create accurate and updated datasets in each country and to decide how best to use the disparate data to determine which areas needed no further mapping and which remained as “gaps” that would require further attention.

The WHO/AFRO NTD database used for this study was built on earlier efforts to standardize the capture of mapping data using digital tools and a small number of linked databases to manage this information.^12^ The database had initially been developed with data fields that recorded district, site, GPS coordinates, number of people tested, age-groups, number of positives, specific NTD, and diagnostic methods, and it had three main levels:

1. an individual (village)-level database
2. an aggregated table, with site-level (subdistrict) data either already available from the countries or aggregated subsequently from their individual-level data
3. a normalized table of data aggregated at the IU (district) level—important because for every district, the presence or absence of each PC-NTD needed to be defined because most NTD interventions are implemented at the district level

At the beginning of the accelerated mapping project, data aggregated at the site level were requested from the national programs, and these were then compiled into a master file of IU (district)-level data. Subsequently, as individual-level data became available, it was aggregated to site-level data that then replaced the initial site-level data, after approval by the countries.

For all mapping survey data acquired during the mapping project, standardized individual data collection forms were used to capture the information required to classify each IU according to WHO endemicity categories and their treatment implications. In addition, all targeted countries were offered the use of an electronic data collection tool (LINKS).^13^ For reasons of time constraints or concerns about the use of cloud-based tools, only 16 countries in the WHO/AFRO NTD Mapping Project used this tool; the other countries opted for paper-based, standardized data collection forms.

After its collection, the mapping data were shared by national programs with the WHO regional data managers who performed completeness and consistency checks. These data managers supported and liaised closely with the national programs to ensure that standard data formats were used and that data were complete, clean, and internally consistent. All mapping data were housed and maintained in the database at WHO/AFRO headquarters in Brazzaville. An interactive portal was initiated with dashboards designed to provide data access to countries and stakeholders that included the mapping results, treatment activities, and progress toward the control and elimination of the NTDs. It was this NTD Mapping Portal that subsequently became the basis of the Expanded Special Program for Elimination of NTD (ESPEN) Portal (see in the following text).

## RESULTS

### Defining the neglected tropical disease “mapping gap.”

At the start of the accelerated mapping project, the enormity of this PC-NTD mapping challenge can be seen in the data of Table 1. Whereas the numbers of districts in each country (and their geographic boundaries) can change over time, still national program managers were able to identify, both from historical and more recently acquired data, which of their districts in 2013 were endemic for each of the 5 PC-NTDs and which still required mapping (i.e., had a “mapping gap”). In many of the districts, more than one disease needed to be mapped, but there were at least 2,200 different districts—of the 4,851 districts in WHO/AFRO in 2013—where mapping would be required.

**Table 1:** Recognized baseline mapping gaps—Pre-mapping project

AFRO	Lymphatic filariasis	Oncho	Schistosomiasis	Soil-transmitted helminthiasis	Trachoma
No. countries with disease-specific mapping gap	18	13	27	27	26
No. districts with disease-specific mapping gap	662	398	981	1,077	1,718

**Figure 1.**
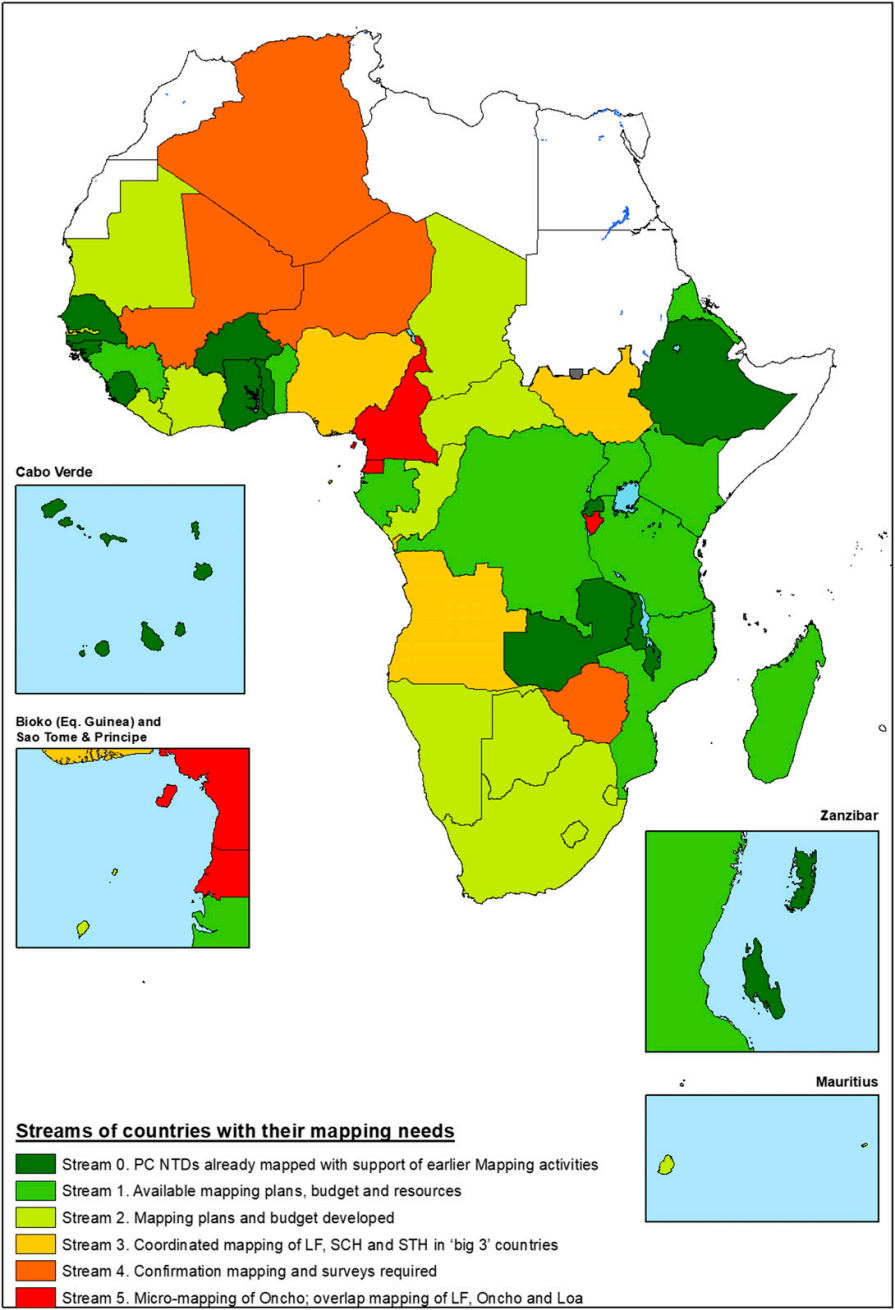
Streams of countries with their mapping needs. Stream 0 (13 countries), stream 1 (10 countries), stream 2 (15 countries), stream 3 (three countries), stream 4 (four countries), and stream 5 (three countries).

Based on the 2013 estimates of mapping needs (Table 1), AFRO’s 47 countries were grouped into six categories according to their perceived mapping needs—one group of countries where standard mapping for the PC-NTDs was thought to be complete already, based on the compiled historical data, and five “streams” of countries at different stages of preparedness for PC-NTD mapping (Figure 1). Then, what followed during the 3 years of the accelerated mapping project (2014–2016) was the progressive roll-out of a large-scale mobilization involving six principal mapping survey teams organized from the WHO/AFRO offices in Ouagadougou, Libreville, Harare, and Brazzaville. These teams were responsible for planning, training, and deploying technical supervisory teams and national recruits to carry out WHO-standardized mapping surveys for the PC-NTDs in 28 different countries.

### Shrinking the gap—Identifying country-specific prevalence of PC-NTDs in Africa.

**Figure 2.**
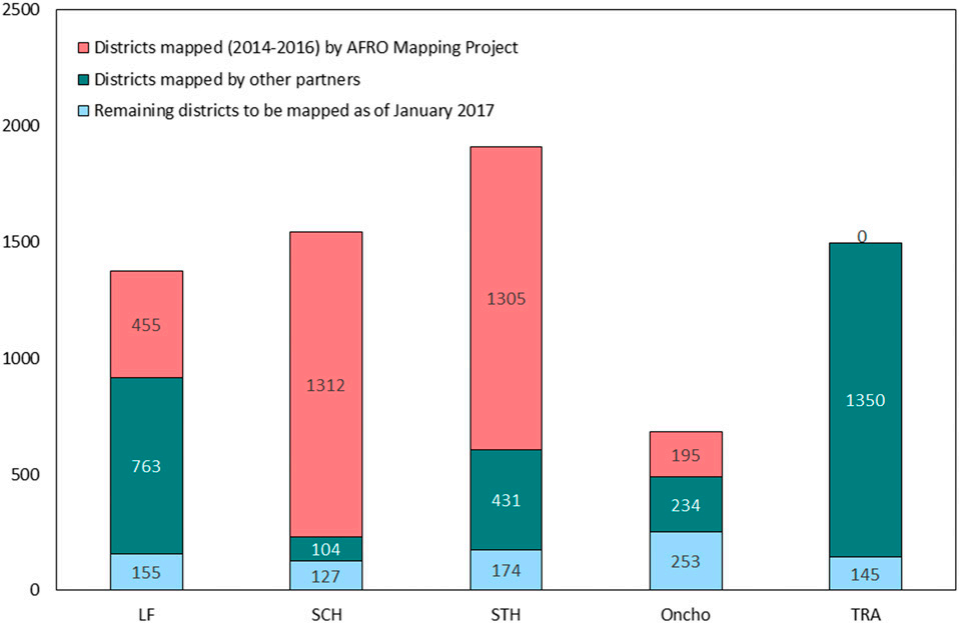
District mapping progress by neglected tropical diseases (2014–2016).

After the initial activities to organize national data that were already available, the mapping teams set out to face the mapping-gap challenges identified in the data of Table 1 and Figure 1. Using the agreed tools and strategies during the 3 years from the beginning of the accelerated NTD mapping project in January 2014 through its end in 2016, the teams carried out mapping for one or more PC-NTDs through almost 6,000 individual surveys in 37 African countries (Table 2) that included a minimum of 2,500 different districts. Despite discovering some districts as endemic for PC-NTDs that had not been recognized as such in the earlier “baseline” evaluations of Table 1, still, by the end of 2016, the WHO-required mapping for the targeted NTDs was complete in all but a few hundred of WHO/AFRO’s 4,851 districts (Figure 2), with more than half of these remaining districts being in just three partially mapped countries. Details of these country-specific NTD prevalence results—both maps and district-level data—can be found in AFRO’s ESPEN Mapping Portal (http://espen.afro.who.int/countries).

**Table 2:** Countries mapped for different NTDs during the AFRO mapping project

NTD	Lymphatic filariasis	Soil-transmitted helminthiasis	Schistosomiasis	Oncho	Trachoma
Countries	Botswana	Angola	Angola	Angola	Benin
	Chad	Benin	Benin	Chad	Burundi
	Congo	Botswana	Botswana	Congo	Cameroon
	Cote d’Ivoire	Burundi	Burundi	Côte d’Ivoire	CAR
	DRC	Chad	Chad	DRC	Chad
	Eritrea	Côte d’Ivoire	Côte d’Ivoire	Eq. Guinea	Congo
	Ethiopia	DRC	DRC	Ethiopia	DRC
	Eq. Guinea	Eritrea	Eritrea	Gabon	Eritrea
	Gabon	Ethiopia	Ethiopia	Nigeria	Ethiopia
	Madagascar	Gabon	Gabon	Tanzania	Gabon
	Mauritania	Gambia	The Gambia		Gambia
	Niger	Guinea	Guinea		Guinea
	Sao Tome and Principe	Kenya	Kenya		Kenya
	South Sudan	Lesotho	Lesotho		Malawi
	Zimbabwe	Liberia	Liberia		Mozambique
		Madagascar	Madagascar		Nigeria
		Mauritania	Mauritania		Senegal
		Mauritius	Mauritius		South Sudan
		Namibia	Namibia		Tanzania
		Nigeria	Nigeria		Uganda
		Seychelles	Seychelles		Zambia
		South Africa	South Africa		Zimbabwe
		South Sudan	South Sudan		
		Swaziland	Swaziland		
		Zimbabwe	Zimbabwe		
# with at least 1 NTD mapped					
37	15	25	25	10	22

DRC = Democratic Republic of the Congo; NTD = neglected tropical disease.

## DISCUSSION

The impact of the work of AFRO’s accelerated NTD Mapping Project was very broad, including:

1. defining PC-NTD disease prevalence in the African region
2. energizing the national NTD programs
3. strengthening national capacity for disease mapping and implementation
4. stimulating operational research for critical tools and strategies for national NTD programs
5. ensuring national ownership of epidemiologic data in coordination with the WHO
6. laying the groundwork for subsequent creation of AFRO’s ESPEN

### Preventive Chemotherapy-neglected tropical disease prevalence.

The remarkable progress in PC-NTD mapping during the 3-year life of the accelerated AFRO–NTD Mapping Project can be appreciated in both Figure 2 and the data found online in AFRO’s ESPEN Mapping Portal (http://espen.afro.who.int/countries). Two principal reasons account for this success: 1) the effectiveness of the mapping project in its close individual working relationship with each country in the African region and 2) the genuinely collaborative public–private partnership supporting the project. The comprehensiveness and standardization of the project’s mapping strategy, data collection, and data management required numerous technical meetings where national program managers, regional and international consultants, and WHO/AFRO technical staff all met to examine and organize the available datasets—one country at a time. Although an intensive, laborious undertaking, this effort proved to be an essential investment in establishing the framework for the rest of the mapping project and a key to its success. Similarly, the comprehensiveness of the project’s goal to define the scope of PC-NTDs in the African region encouraged a broad partnership among donors who recognized the opportunities to support individual mapping initiatives that would be complementary to each other, ensuring that the result would be a practical framework underlying the very broad goal of NTD control and elimination in Africa.

### Energizing national NTD programs.

In the years leading up to 2014, most African countries had been encouraged and supported by WHO/AFRO to create comprehensive national 5-year Master Plans for their NTD programs.^14^ All of these plans called for “mapping” each of the PC-NTDs, and, indeed, much of the rest of the master plan activity depended on the results of this mapping to target needed program implementation. Without resources to carry out the necessary mapping, progress against the NTDs was often stymied, or at least severely constrained. It was fortunate, therefore, that because of the decision by the BMGF to invest in stimulating an accelerated WHO/AFRO NTD Mapping Project, funds became available that allowed many of the proposed, but stalled, master plan activities to be jump-started quickly.

Although it is easy to appreciate how such funding would directly stimulate the mapping and master plan activities, there was another—probably equally important—consequence. The very engagement of the BMGF in investing to support an accelerated, comprehensive African NTD mapping initiative leveraged an almost equivalent amount of funding from other donors as well, thereby ensuring both that the mapping could be completed for all five PC-NTDs and that the WHO/AFRO’s entire program targeting the NTDs would be significantly energized.

### National neglected tropical disease capacity building.

Implementation of the WHO-standardized mapping began with field training for national survey teams recruited by the NTD programs to conduct the necessary surveys. These surveyors were generally laboratory technicians (tasked to carry out the diagnostic test or procedure), nurses (tasked to draw blood), and medical doctors (as supervisors). Before the fieldwork, technical training was conducted to review the survey site, population to be tested, sample collected, diagnostic used, test interpretation, and method/platform for data collection and recording. New diagnostic tools (e.g., CCA for SCH) or strategies (e.g., “confirmatory mapping” for LF) were given special attention during the training. As a result of their engagement in the accelerated NTD Mapping Project, these trainees now constitute a pool of technically skilled resources that countries can use for the program assessments and epidemiologic analyses so essential for both their NTD and other public health initiatives.

### Operational research to refine mapping tools and strategies.

The WHO/AFRO’s accelerated NTD Mapping Project, in addition to defining where and in what measure PC-NTDs could be found in 37 countries, also provided unique opportunities to evaluate newly developed diagnostic tools and mapping strategies. Indeed, at least 25 of the 37 countries in the mapping project participated in this operational research. Most of the studies compared operationalization of newer and older diagnostic tools—for SCH, onchocerciasis, STH, and LF—but some also tested strategies for integrated mapping approaches and for confirmation of earlier mapping results.^15^ In addition, even after the mapping project ended, numerous research studies (both mapping- and diagnostics-related) based on this mapping project work have been initiated, including important studies focused on elimination mapping for onchocerciasis,^9^ and the still-unresolved, most effective diagnostic algorithms to safely treat onchocerciasis in areas co-endemic with loiasis.^16^

### Preparing for the future—ESPEN: The Expanded Special Program for Elimination of NTDs.

At the close in 2015 of the 20-year-old APOC program that oversaw national onchocerciasis control and other NTD activities in Africa, a new organization, ESPEN, was formed in 2016 to fill APOC’s earlier roles and to consolidate the WHO/AFRO NTD initiatives. In 2016, because the infrastructure initially developed for the accelerated mapping project was so relevant to the needs of this new program, critical components necessary for the new program were immediately available, including

1. personnel skilled in the epidemiologic assessment of NTDs
2. a roster of consultants (mostly national experts) available to deploy to countries in Africa to collate available mapping data, identify remaining mapping gaps, and plan and implement surveys where necessary—all using uniform, standardized techniques
3. technical partners to ensure quality and standardization across surveys
4. training materials, field manuals, and SOPs—all reflecting the newest diagnostic tools and insightful strategies for conducting epidemiologic and mapping surveys
5. established supply chain processes for diagnostics and ancillary reagents and supplies

Of particular value to the ESPEN was the mapping project’s database and portal, which was transferred and built rapidly into an ESPEN portal that was live online only a few months after the ESPEN was established in 2016. That portal (http://espen.afro.who.int/countries) has provided the first WHO subnational-level repository of data and cartography of disease endemicity and treatment activities. Forty-three countries soon agreed to make their countries’ data publicly available, and, indeed, by 2019, this portal had become the go-to site for African data on the NTDs—with more than 4,000 maps and corresponding datasets openly available to the public. In 2019 alone, 9,255 users visited the portal from 153 countries during 19,362 sessions. The ESPEN has continued working with the former staff of the WHO/AFRO NTD Mapping Project and its partners to ensure a continuity of data management built not only on a consolidation of the collected data but also, especially, on the trusted relationships that the mapping project had established with the national NTD programs.
